# Adverse childhood experiences and neurodevelopmental disorder traits: A nationwide cross‐sectional study

**DOI:** 10.1002/pcn5.70397

**Published:** 2026-08-24

**Authors:** Hiroki Yamada, Toshinori Nakamura, Tomonari Yoshizawa, Kazuhiro Suzuki, Daimei Sasayama, Hideo Honda, Takahiro Tabuchi, Shinsuke Washizuka

**Affiliations:** ^1^ Department of Psychiatry Shinshu University School of Medicine Nagano Japan; ^2^ Department of Community Mental Health Shinshu University School of Medicine Nagano Japan; ^3^ Department of Child and Adolescent Developmental Psychiatry Shinshu University School of Medicine Nagano Japan; ^4^ Division of Epidemiology, School of Public Health, Graduate School of Medicine Tohoku University Miyagi Japan

**Keywords:** adverse childhood experience, attention‐deficit/hyperactivity disorder, autism spectrum disorder, developmental disorder

## Abstract

**Aim:**

In this study, we aimed to examine the associations between adverse childhood experience (ACE) exposure, adult autism spectrum disorder (ASD), and attention‐deficit/hyperactivity disorder (ADHD) traits in a nationwide sample, and assess links with specific symptom subdomains.

**Methods:**

We conducted a cross‐sectional analysis using 2024 data from the Japan “Society and New Tobacco” Internet Survey. Adults (*N* = 29,268; ≥18 years) completed self‐reports including the 10‐item ACE questionnaire (categorized: 0, 1, 2, 3, or ≥4 ACEs), Autism‐Spectrum Quotient short form, and Adult ADHD Self‐Report Scale. Logistic regression models assessed associations between ACEs and positive ASD (score ≥ 7) and ADHD (score ≥ 4) screens, adjusting for demographics, health, mental distress (K6), and alcohol use.

**Results:**

Among respondents, 4.9% reported experiencing ≥4 ACEs. Positive screening rates were 11.9% for ASD traits and 7.7% for ADHD traits. ASD traits were only associated with high ACE exposure (≥4 ACEs: odds ratio [OR] 1.29, 95% confidence intervals [CIs] 1.11–1.49). ADHD traits showed a dose–response relationship starting at 1 ACE (OR 1.15, 95% CI 1.00–1.31), and increased with greater exposure (≥4 ACEs: OR 1.70, 95% CI 1.45–1.98). High exposure was linked to specific ASD symptoms (e.g., social and communication difficulties) and ADHD symptoms (e.g., disorganization, task avoidance, and hyperactivity).

**Conclusion:**

ACEs were associated with adult ASD and ADHD traits and related symptom subdomains in adulthood.

## INTRODUCTION

Adverse childhood experiences (ACEs), encompassing various forms of abuse, neglect, and household dysfunction occurring before age 18 years, are a pervasive public health issue with profound, lifelong consequences for children's health and well‐being.^1^, ^2^, ^3^ Exposure to ACEs is remarkably common; approximately 61% of adults have experienced ≥1 ACE, and about one in six (16%) have experienced ≥4 distinct categories of adversity.^4^ An ACE study by Felitti et al.^5^ established a foundational principle in this field: the impact of childhood adversity is cumulative and dose‐dependent.^6^, ^7^, ^8^, ^9^ Their work revealed a strong, graded relationship between the number of ACEs and risk of negative health outcomes in adulthood, including depression, substance use disorders, and chronic illnesses such as heart disease and cancer.^5^, ^10^, ^11^, ^12^ This cumulative, dose‐dependent risk underscores the importance of viewing the ACE score as a count of events and a proxy for the degree of an individual's developmental disruption.

Recently, neurodevelopmental conditions such as autism spectrum disorder (ASD) and attention‐deficit/hyperactivity disorder (ADHD) have been increasingly recognized to persist across the lifespan, instead of being confined to childhood. ASD is characterized by persistent difficulties in social communication and interaction, along with restricted and repetitive patterns of behavior or interests.^13^ In Japan, the estimated 5‐year cumulative incidence of ASD in children born between fiscal years 2009 and 2014 is 2.75%,^14^ indicating an increasing ASD diagnostic incidence rate. ADHD involves a persistent pattern of inattention and/or hyperactivity‐impulsivity that impairs functioning, with a global pooled prevalence of 5%–7% in children.^15^ Although these conditions have distinct core features, clinical challenges arise due to symptomatic overlap with childhood trauma sequelae. For instance, trauma‐related behaviors such as social withdrawal, emotional dysregulation, and avoidance of eye contact can resemble the social communication deficits seen in ASD.^16^, ^17^, ^18^, ^19^, ^20^, ^21^, ^22^ Similarly, hypervigilance, restlessness, and distractibility associated with post‐traumatic stress closely mimic ADHD symptoms, leading to possible misdiagnosis.^23^, ^24^, ^25^ Trauma, as an environmental exposure, produces behaviors that imitate conditions with distinct neurodevelopmental origins. Distinguishing between these overlapping presentations is essential for accurate diagnosis and effective intervention.^26^, ^27^, ^28^

Although a growing body of research highlights a significant association between ACEs and neurodevelopmental conditions, our understanding of this relationship remains incomplete. Three critical gaps persist. First, whether the dose–response principle of ACEs applies uniformly across different domains of psychopathology remains unclear. Second, prior research has focused on global symptom scores, leaving it uncertain which specific behavioral manifestations are most strongly linked to a history of trauma. Whether ACEs exert a diffuse effect or selectively target particular domains remains underexplored. Finally, diagnostic overlap raises a clinically relevant question: “Which aspects of trauma‐related symptoms and neurodevelopmental traits are associated, and how do they differ?” Clarifying the associations and distinctions between them may help clinicians differentiate these presentations in practice.

To address these questions, we conducted a cross‐sectional analysis of a large, nationwide sample of Japanese adults (*N* = 29,268). Using this dataset, ACEs' role as a mediating factor in the association between ADHD symptoms and severe psychological distress has been suggested.^29^ This dataset allowed us to examine dose–response relationships between cumulative ACE exposure and self‐reported ASD and ADHD traits in adulthood using validated screening tools: the Autism‐Spectrum Quotient short form (AQ‐J‐10) and Adult ADHD Self‐Report Scale (ASRS). This study aimed to (1) characterize and compare the dose–response relationships linking ACEs to adult ASD and ADHD traits; (2) identify specific symptom domains within each trait profile most strongly associated with high ACE exposure; and (3) explore evidence for trauma‐related presentations that may mimic, yet differ from, classic neurodevelopmental profiles.

## METHODS

### Study design and participants

This study employed a cross‐sectional design using data from the 2024 wave of the Japan “Society and New Tobacco” Internet Survey (JASTIS), a large, nationally representative online research cohort established in 2015 to investigate health and social factors in the Japanese population.^30^ Although JASTIS is an ongoing longitudinal internet cohort study, the present analysis used only the 2024 wave because measures of ACEs, AQ‐J‐10, and ASRS were simultaneously available in that survey wave. Therefore, this study was designed as a cross‐sectional analysis examining associations between retrospectively reported ACEs and current neurodevelopmental traits screening scores in adulthood.

**Figure 1:**
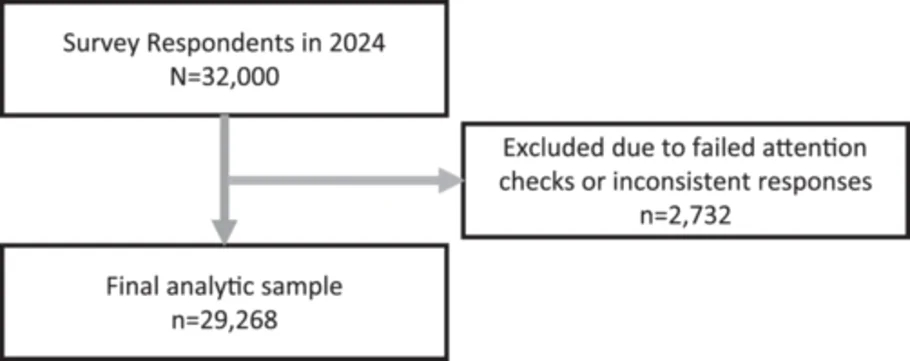
Participant flowchart.

Multiple studies on ACEs have been published using JASTIS data, and the sampling methodology has been reported elsewhere.^31^ Participants were recruited through stratified quota sampling from a major internet research panel to approximate the demographic distribution of the general adult population across Japan's 47 prefectures. Invitations were sent to panelists, and those who provided informed consent completed a web‐based questionnaire. In the 2024 survey wave, 32,000 adults aged 18 years or older participated. To ensure data quality, the survey included attention‐check items and algorithms to detect random or inconsistent responses; individuals failing these checks were excluded from analysis. After excluding these respondents, the final analytic sample comprised 29,268 participants. A participant flowchart describing inclusion and exclusion procedures is presented in Figure 1. The Institutional Review Board of Tohoku University approved all procedures (approval number: 2024‐1‐517).

### Measures

#### ACEs

ACEs were assessed using a 10‐item questionnaire adapted from the original Centers for Disease Control and Prevention‐Kaiser ACE Study.^5^ Respondents reported whether they had experienced each of the following before 18 years of age: emotional abuse, physical abuse, sexual abuse, emotional neglect, physical neglect, parental divorce or separation, domestic violence in the household, household substance abuse, household mental illness, and incarceration of a household member. Each “yes” response was scored as 1, and a total ACE score (range: 0–10) was calculated by summing the items. Consistent with prior research, ACE exposure was categorized into five groups: 0 (no ACEs; reference group), 1, 2, 3, and ≥4 ACEs.^4^, ^5^ This categorical approach captures the cumulative effect of adversity and aligns with prior research identifying ≥4 ACEs as a high‐risk threshold. The questionnaire was administered in Japanese; a validated Japanese version of this ACE scale has been used in previous epidemiological studies and demonstrated good reliability.^32^

#### ASD traits

ASD traits were measured using the 10‐item short form of the AQ‐J‐10^33^—a brief, validated screening tool derived from the original 50‐item scale, which demonstrated acceptable internal consistency and sensitivity in Japanese populations. Each item presents a statement to which respondents indicated their agreement. A cutoff score ≥ 7 was used to identify individuals with significant ASD traits.^33^ The AQ‐J‐10 assesses key domains: social skills, communication, imagination, attention switching, and attention to detail. Three items are reverse scored (i.e., “I prefer to do things with others rather than on my own,” “I would rather go to the theater than to a museum,” and “I find it easy to work out what someone is thinking or feeling just by looking at their face”), where agreement indicates fewer autistic traits. For descriptive analyses, we recorded the total AQ‐J‐10 score and whether it was ≥7 (indicating a high burden of autistic traits). To explore specific symptom patterns, each AQ‐J‐10 item (binary coded as indicative or not indicative of an ASD trait) was also analyzed individually in relation to ACEs.

#### ADHD traits

ADHD traits were assessed using the six‐item version of the ASRS Screener.^34^ The Japanese version has demonstrated good reliability and validity in prior studies.^35^ Following standard scoring guidelines, respondents were considered ASRS‐positive if they endorsed at least four of six items, optimizing sensitivity and specificity for identifying likely ADHD cases. The ASRS items capture symptoms across inattentive and hyperactive‐impulsive domains over the past 6 months. As with ASD traits, we analyzed the dichotomous outcome of screening positive for ADHD (ASRS ≥ 4) and each item individually.

### Covariates

Several covariates known to be associated with ACE exposure and/or neurodevelopmental traits were included to control for potential confounding. Mental health status was measured using the Kessler 6 (K6) scale, a six‐item instrument assessing non‐specific psychological distress; scores ≥ 5 indicate moderate mental distress.^36^ The K6 scale has been validated in Japan and captures symptoms of depression and anxiety that may influence self‐reported traits.^37^

Alcohol use was assessed using the Alcohol Use Disorders Identification Test, a 10‐item screening tool for hazardous drinking; scores ≥ 8 suggest at‐risk alcohol use.^38^, ^39^

Sociodemographic covariates included age (years), sex (male or female), highest educational attainment (university graduate vs. less), marital status (married, never married, divorced, or widowed), annual household income (<4, 4–10, or ≥10 million Japanese Yen), employment status (employed vs. not employed), home ownership (homeowner vs. non‐homeowner), and the presence of any chronic physical illness (yes/no). These variables were self‐reported and selected in accordance with previous literature.^31^ By adjusting for this broad set of covariates, we isolated the association between ACEs and ASD/ADHD traits from potential confounding influences.

### Statistical analysis

Descriptive statistics were first computed to characterize the sample's demographics, ACE distribution, and prevalence of ASD and ADHD traits. We then conducted multivariable logistic regression analyses to examine the association between ACE exposure and neurodevelopmental traits. Two primary outcomes were used: (1) a positive screen for ASD traits (AQ‐J‐10 ≥ 7) and (2) for ADHD traits (ASRS ≥ 4). For each outcome, a logistic regression model was fitted with ACE category as the key independent variable, entered as four dummy variables (1, 2, 3, and ≥4 ACEs), using 0 ACEs as the reference group. All models were adjusted for the covariates described above. Adjusted odds ratios (aORs) with 95% confidence intervals (CIs) were calculated to quantify the strength of association between ACE exposure and each outcome. To identify the specific symptom domains contributing to these associations, we conducted item‐level logistic regressions for each AQ‐J‐10 and ASRS item, using ACE ≥ 4 (vs. <4) as the predictor—selected based on our primary findings to represent high ACE exposure. These models were also adjusted for all covariates. All analyses were conducted using R version 4.2.2 (Posit Software, PBC, Boston, MA, USA). To ensure reproducibility, all analyses were independently replicated by Yoshizawa and Suzuki. Statistical significance was set at *p* < 0.05 (two‐tailed) for all primary outcomes.

## RESULTS

Among the 29,268 participants, 4.9% (*n* = 1462) reported experiencing ≥4 ACEs. The prevalence of ASD traits, as measured by the AQ‐J‐10, was 11.9% (*n* = 3503), whereas the prevalence of ADHD traits, as measured by the ASRS, was 7.7% (*n* = 2269; Table 1).

**Table 1:** Participant demographics (*N* = 29,268).

	Number (%)
AQ‐J‐10 (cutoff 7)	3503 (11.9%)
ASRS (cutoff 4)	2269 (7.7%)
ACE0	21,917 (74.8%)
ACE1	3682 (12.5%)
ACE2	1384 (4.7%)
ACE3	823 (2.8%)
ACE4+	1462 (4.9%)
AQ‐J‐10	
Like to do things with others	18,522 (63.2%)
Say impolite things naively	6876 (23.4%)
Tend to have strong interests	8520 (29.1%)
Hard to know others' intentions	7206 (24.6%)
Prefer a theater to museum	15,856 (54.1%)
Often the last to understand a joke	8112 (27.7%)
Easy to work out other's thinking	15,152 (51.7%)
Like categories of things	14,419 (49.2%)
Hard to imagine to be someone else	8068 (27.5%)
Hard to work out other's intention	9062 (30.9%)
ASRS	
Trouble wrapping up final details of a project	9664 (33.0%)
Difficulty getting things in order when doing a task	8193 (27.9%)
Problems remembering appointments or obligations	7187 (24.5%)
Avoiding or delaying getting started on tasks	2776 (9.4%)
Fidgeting or squirming with hands or feet when sitting	2538 (8.6%)
Feeling overly active and compelled to do things	1789 (6.1%)
ACE	
Emotional abuse	2641 (9.0%)
Physical abuse	2133 (7.2%)
Sexual abuse	1176 (4.0%)
Emotional neglect	2446 (8.3%)
Physical neglect	879 (3.0%)
Parental divorce	2914 (9.9%)
Intimate partner violence	1377 (4.7%)
Substance abuse	1134 (3.8%)
A parent with a mental illness	1454 (4.9%)
Incarcerated household member	453 (1.5%)
Confounding factors	
K6 (cutoff 5)	12,485 (42.6%)
AUDIT (cutoff 8)	4027 (13.7%)
SEX (MAN)	14,452 (49.3%)
Marital status	
Single	9356 (31.9%)
Married	17,194 (58.7%)
Widowed	897 (3.0%)
Divorced	1821 (6.2%)
Smokers	5037 (17.2%)
University graduation	15,059 (51.4%)
Houseowner	19,473 (66.5%)
Age	
10s	521 (1.7%)
20s	5043 (17.2%)
30s	4825 (16.4%)
40s	5037 (17.2%)
50s	4621 (15.7%)
60s	4485 (15.3%)
70s	4123 (14.0%)
80s	613 (2.0%)
Employed	19,952 (68.1%)
Chronic disease^a^	10,273 (35.0%)
Household income	
Low income^b^	11,096 (37.9%)
Middle income^b^	14,980 (51.1%)
High income^b^	3192 (10.9%)

*Note*: Data are presented as *n* (%).

Abbreviations: ACE, adverse childhood experience; AQ‐J‐10, Autism‐Spectrum Quotient short form; ASRS, Adult attention‐deficit/hyperactivity disorder Self‐Report Scale; K6, Kessler 6.

^a^ Participants with at least one chronic disease, including hypertension, angina pectoris/myocardial infarction, cerebral infarction/cerebral hemorrhage, diabetes mellitus, dyslipidemia, pneumonia, bronchitis, asthma, chronic obstructive pulmonary disease, chronic kidney disease, chronic hepatitis/liver cirrhosis, immunological disorders, cancer/malignant neoplasms, epilepsy, and mental disorders other than depression and alcohol dependence.

^b^ Household income was categorized as low income (≤4 million yen), middle income (4.01–10 million yen), or high income (≥10.01 million yen).

Logistic regression analyses, adjusted for potential confounders—including mental health status, substance use, and demographic and socioeconomic factors—revealed a positive association between cumulative ACE exposure and ASD and ADHD traits. Among individuals with ≥4 ACEs, the aOR for exhibiting ASD traits was 1.29 (95% CI: 1.11–1.49), indicating a significant association. For ADHD traits, significant associations were observed even at lower levels of ACE exposure; individuals with ≥1 ACEs had an aOR of 1.15 (95% CI: 1.00–1.31), suggesting that ADHD traits may be more sensitive to ACE exposure than ASD traits (Tables 2 and 3).

**Table 2:** Logistic regression analysis for autism spectrum disorder (ASD) traits.

	aOR (95% CI)	*p*‐value
ACE0	1	
ACE1	0.93 (0.82–1.04)	0.208
ACE2	1.10 (0.94–1.29)	0.211
ACE3	1.06 (0.86–1.29)	0.535
ACE4+	1.29 (1.11–1.49)	<0.001
K6 (cutoff 5)	2.17 (2.00–2.35)	<0.001
AUDIT (cutoff 8)	1.04 (0.94–1.15)	0.405
SEX (MAN)	1.84 (1.70–2.00)	<0.001
Single	1	
Married	0.87 (0.79–0.95)	0.003
Widowed	0.71 (0.50–0.97)	0.04
Divorced	0.96 (0.81–1.14)	0.711
Smokers	0.96 (0.87–1.06)	0.501
University graduation	0.92 (0.85–0.99)	0.042
Houseowner	1.08 (1.00–1.17)	0.045
10s	1	
20s	0.74 (0.59–0.94)	0.011
30s	0.62 (0.49–0.79)	<0.001
40s	0.50 (0.40–0.64)	<0.001
50s	0.44 (0.35–0.57)	<0.001
60s	0.31 (0.24–0.41)	<0.001
70s	0.27 (0.21–0.36)	<0.001
80s	0.16 (0.10–0.27)	<0.001
Employed	1.07 (0.97–1.18)	0.166
Chronic disease	1.12 (1.02–1.22)	0.009
Low income	1	
Middle income	0.85 (0.78–0.93)	<0.001
High income	0.79 (0.68–0.90)	<0.001

*Note*: The model was adjusted for all other variables listed in the table. The dependent variable is a positive screen for ASD traits (Autism‐Spectrum Quotient short form [AQ‐J‐10] score ≧ 7). The reference groups are the following: ACE0 for ACE categories, single for marital status, 10s for age, and low income for household income.

Abbreviations: ACE, adverse childhood experience; aOR, adjusted odds ratio; CI, confidence interval; K6, Kessler 6.

**Table 3:** Logistic regression analysis for attention‐deficit/hyperactivity disorder (ADHD) traits.

	aOR (95% CI)	*p*‐value
ACE0	1	
ACE1	1.15 (1.00–1.31)	0.038
ACE2	1.35 (1.12–1.62)	<0.001
ACE3	1.53 (1.23–1.88)	<0.001
ACE4+	1.70 (1.45–1.98)	<0.001
K6 (cutoff 5)	5.03 (4.47–5.67)	<0.001
AUDIT (cutoff 8)	1.46 (1.29–1.64)	<0.001
SEX (MAN)	1.27 (1.15–1.41)	<0.001
Single	1	
Married	1.02 (0.91–1.14)	0.624
Widowed	1.23 (0.77–1.89)	0.352
Divorced	1.10 (0.87–1.37)	0.384
Smokers	1.00 (0.88–1.13)	0.952
University graduation	1.12 (1.02–1.24)	0.015
Houseowner	1.07 (0.97–1.18)	0.155
10s	1	
20s	0.70 (0.55–0.90)	0.005
30s	0.50 (0.38–0.65)	<0.001
40s	0.37 (0.28–0.48)	<0.001
50s	0.22 (0.17–0.29)	<0.001
60s	0.11 (0.08–0.15)	<0.001
70s	0.07 (0.05–0.11)	<0.001
80s	0.08 (0.03–0.16)	<0.001
Employed	0.96 (0.85–1.09)	0.556
Chronic disease	1.28 (1.15–1.43)	<0.001
Low income	1	
Middle income	0.89 (0.80–0.99)	0.033
High income	0.89 (0.75–1.05)	0.201

*Note*: The model was adjusted for all other variables listed in the table. The dependent variable is a positive screen for ADHD traits (Adult ADHD Self‐Report Scale [ASRS] score ≥ 4). The reference groups are the following: ACE0 for ACE categories, single for marital status, 10s for age, and low income for household income.

Abbreviations: ACE, adverse childhood experience; aOR, adjusted odds ratio; CI, confidence interval; K6, Kessler 6.

Examination of individual AQ‐J‐10 and ASRS items revealed distinct patterns of association with ACE exposure. For ASD traits, higher ACE scores were positively associated with difficulties in social appropriateness (e.g., “say impolite things naively”; OR = 1.19, 95% CI: 1.04–1.36) and humor comprehension (“often the last to understand a joke”; OR = 1.17, 95% CI: 1.03–1.33). Notably, higher ACE exposure was associated with less frequent endorsement of the item “easy to work out others' thinking” (OR = 0.78, 95% CI: 0.69–0.87). For ADHD traits, the strongest associations with ACEs were observed for difficulties in task organization (“difficulty getting things in order when doing a task”; OR = 1.25, 95% CI: 1.09–1.45), task initiation (“avoiding or delaying getting started on tasks”; OR = 1.36, 95% CI: 1.17–1.59), and hyperactivity (“feeling overly active and compelled to do things”; OR = 1.43, 95% CI: 1.21–1.70; Table 4).

**Table 4:** Logistic regression analysis for high adverse childhood experience (ACE) exposure.

	aOR (95% CI)	*p*‐value
AQ‐J‐10		
I prefer to do things with others rather than on my own.	1.14 (1.01–1.28)	0.031
Other people frequently tell me that what I have said is impolite, even though I think it is polite.	1.19 (1.04–1.36)	0.007
I tend to have very strong interests, which I get upset about if I can't pursue.	1.20 (1.05–1.35)	0.004
When I am reading a story, I find it difficult to work out the characters' intentions.	0.92 (0.80–1.05)	0.235
I would rather go to the theater than to a museum.	1.09 (0.97–1.22)	0.129
I am often the last to understand the point of a joke.	1.17 (1.03–1.33)	0.015
I find it easy to work out what someone is thinking or feeling just by looking at their face.	0.78 (0.69–0.87)	<0.001
I like to collect information about categories of things (e.g., types of cars, birds, trains, and plants).	1.11 (0.98–1.24)	0.074
I find it difficult to imagine what it would be like to be someone else.	1.10 (0.96–1.26)	0.155
I find it difficult to work out people's intentions.	1.04 (0.90–1.19)	0.566
ASRS		
How often do you have trouble wrapping up the final details of a project, once the challenging parts have been done?	0.97 (0.84–1.11)	0.678
How often do you have difficulty getting things in order when you have to do a task that requires organization?	1.25 (1.09–1.45)	0.001
How often do you have problems remembering appointments or obligations?	1.06 (0.93–1.21)	0.357
When you have a task that requires a lot of thought, how often do you avoid or delay getting started?	1.36 (1.17–1.59)	<0.001
How often do you fidget or squirm with your hands or feet when you have to sit down for a long time?	1.08 (0.91–1.27)	0.328
How often do you feel overly active and compelled to do things, like you were driven by a motor?	1.43 (1.21–1.70)	<0.001
K6 (cutoff 5)	2.51 (2.18–2.89)	<0.001
AUDIT (cutoff 8)	2.14 (1.87–2.44)	<0.001
SEX (MAN)	0.66 (0.58–0.75)	<0.001
Single	1	
Married	1.09 (0.94–1.25)	0.216
Widowed	1.51 (0.98–2.25)	0.046
Divorced	1.61 (1.29–2.01)	<0.001
Smokers	1.57 (1.37–1.79)	<0.001
University graduation	0.71 (0.63–0.80)	<0.001
Houseowner	0.87 (0.77–0.98)	0.031
10s	1	
20s	0.88 (0.63–1.24)	0.465
30s	0.72 (0.51–1.03)	0.067
40s	0.66 (0.47–0.95)	0.023
50s	0.61 (0.43–0.89)	0.008
60s	0.41 (0.28–0.61)	<0.001
70s	0.21 (0.13–0.33)	<0.001
80s	0.04 (0.006–0.13)	<0.001
Employed	0.84 (0.73–0.97)	0.021
Chronic disease	2.08 (1.85–2.35)	<0.001
Low income	1	
Middle income	0.91 (0.80–1.04)	0.194
High income	1.02 (0.83–1.24)	0.847

*Note*: The model was adjusted for all other variables listed in the table. The dependent variable is high ACE exposure (≥4 ACE). Individual AQ‐J‐10 and ASRS items were entered as predictors.

Abbreviations: aOR, adjusted odds ratio; AQ‐J‐10, Autism‐Spectrum Quotient short form; ASRS, Adult attention‐deficit/hyperactivity disorder Self‐Report Scale; CI, confidence interval; K6, Kessler 6.

## DISCUSSION

The present findings indicate that individuals with greater exposure to ACEs exhibit higher levels of adult ASD and ADHD traits. This is consistent with prior research showing that autistic individuals often experience a greater burden of early‐life adversity,^40^ and childhood trauma is linked to ADHD symptoms in adulthood.^28^

Our results extend this literature by demonstrating that ACEs are associated with general mental health outcomes and specific dimensional traits of neurodevelopmental conditions in adulthood.

Our observations regarding the ACE–ASD trait relationship were in agreement with findings from prior studies.^5^

Regarding ASD, our findings align with evidence that children on the autism spectrum are approximately twice as likely as their peers to have experienced ≥4 ACEs.^41^ It may be that only severe, cumulative trauma—above a certain threshold—meaningfully amplifies autism‐related traits.

Conversely, the association between ACEs and ADHD traits appeared more linear in this sample, suggesting that even lower levels of childhood adversity may contribute to elevated ADHD trait expression in adulthood. Therefore, this difference in dose–response patterns between ASD and ADHD traits is a key and novel finding.

One possible explanation for the differential ACE associations observed in this study is that some “ASD‐like” social and behavioral difficulties among individuals with high ACE exposure may reflect trauma‐related adaptations rather than core neurodevelopmental features. Chronic interpersonal trauma in childhood can alter social cognition; trauma survivors may develop hypermentalizing tendencies—over‐attributing intentions or emotions to others—as a coping mechanism.^42^ This form of hypermentalizing is considered a trauma‐related social‐cognitive adaptation and may manifest as social anxiety, mistrust, or interpersonal difficulties.^43^ This contrasts with the typical social‐cognitive profile of ASD, often marked by underestimation of others' mental states. As such, trauma‐exposed individuals may exhibit behaviors that superficially resemble ASD traits (e.g., social withdrawal or misreading social cues) but stem from hypervigilance and defensive mentalizing. This distinction may help explain the association of high ACE exposure with increased ASD trait scores: the traits detected may reflect trauma‐induced social coping styles rather than core features of ASD. In other words, ACEs may heighten sensitivity to adverse experiences and emotionally charged social stimuli, negative and positive.^44^

This study has some limitations. First, the cross‐sectional design precludes causal inference; we cannot determine whether ACEs directly contributed to elevated ASD or ADHD traits, or whether pre‐existing developmental vulnerabilities increased the likelihood of encountering adversity. Longitudinal data would be necessary to establish temporal directionality. Second, the data were collected via an online self‐report survey, which may introduce self‐selection bias—individuals with certain experiences may have been more likely to participate—and relies on retrospective recall of childhood events, which is subject to memory distortion.

Moreover, ACE exposure and neurodevelopmental traits were self‐reported rather than clinician‐assessed or objectively verified; thereby, they may be influenced by current mood, insight, or social desirability, and may not fully capture clinically diagnosable conditions. These factors could lead to either overestimation or underestimation of true associations. Finally, although the sample was nationwide, it may not fully represent the broader Japanese population, limiting generalizability.

Future research should incorporate prospective designs, clinician‐administered diagnostic assessments, and, where possible, informant reports or biological markers to validate and extend these findings.

## CONCLUSION

ACEs were significantly associated with elevated levels of ASD and ADHD traits in adulthood. Individuals with a history of multiple ACEs were more likely to exhibit the core characteristics of these neurodevelopmental conditions, following adjustment for demographic and mental health factors. Notably, ACEs showed a threshold effect in relation to ASD traits and a dose‐dependent association with ADHD traits. Furthermore, high ACE exposure was associated with several specific ASD‐ and ADHD‐related symptoms, particularly those involving social communication and executive functioning. These findings underscore the importance of integrating trauma history into the assessment and conceptualization of neurodevelopmental disorders. For clinicians, adopting a trauma‐informed approach is essential—routinely screening for ACEs and considering how trauma may shape symptom presentation in individuals with ASD or ADHD traits. For researchers and policymakers, these findings highlight the need for strategies to prevent childhood adversity and for longitudinal studies to further investigate its association with neurodevelopmental traits.

## PERMISSION TO REPRODUCE MATERIAL FROM OTHER SOURCES

Not applicable. All tables and other materials presented in this work are original to the authors.

## DECLARATION OF GENERATIVE AI AND AI‐ASSISTED TECHNOLOGIES IN THE MANUSCRIPT PREPARATION PROCESS

During the preparation of this work, the author(s) used ChatGPT (GPT‐o3, GPT‐4.5, by OpenAI) and Gemini (Gemini 2.5 Pro, by Google) to improve its English readability and entrusted Editage (www.editage.jp) for editing. After using these tools/services, the author(s) reviewed and edited the content as needed and take full responsibility for the content of the published article.

## AUTHOR CONTRIBUTIONS

**Hiroki Yamada**: Conceptualization; methodology; writing—original draft. **Toshinori Nakamura**: Supervision; writing—review and editing. **Tomonari Yoshizawa**: Data analysis; supervision. **Kazuhiro Suzuki**: Data analysis; supervision. **Daimei Sasayama**: Supervision. **Hideo Honda**: Supervision. **Takahiro Tabuchi**: Funding acquisition; supervision. **Shinsuke Washizuka**: Supervision. All the authors have read and approved the final manuscript.

## CONFLICT OF INTEREST STATEMENT

H.Y. and K.S. have no known competing financial interests. T.Y. has received honoraria for lectures from Otsuka Pharmaceutical Co. Ltd. and Sumitomo Pharma Co. Ltd. T.N. has received research grants from Boehringer Ingelheim, and honoraria for lectures from Otsuka Pharmaceutical Co. Ltd., Eisai Co. Ltd., Janssen Pharmaceutical K.K., MSD K.K., Lundbeck Japan, Sumitomo Pharma Co. Ltd., and Shionogi & Co. Ltd. D.S. has received honoraria for lectures from Lundbeck Japan K.K., Otsuka Pharmaceutical Co. Ltd., Takeda Pharmaceutical Co. Ltd., and Shionogi Pharma Co. Ltd. H.H. has received honoraria for lectures from Nobelpharma Co. Ltd., MSD, Shionogi Pharma Co. Ltd., Otsuka Pharmaceutical Co. Ltd., Janssen Pharmaceutical K.K., Sumitomo Parma Co. Ltd., and Lundbeck Japan K.K. T.T. has received financial support for research from Daiichi Sankyo Healthcare Co. Ltd., Johnson & Johnson K.K., Data Seed Inc., Workout‐Plus LLC and EMMA Co. Ltd. S.W. has received research grants from Japan Society for the Promotion of Science, Otsuka Pharmaceutical Co. Ltd., Mochida Pharmaceutical Co. Ltd., Sumitomo Pharma Co. Ltd., and Shionogi & Co. Ltd., and honoraria for lectures from Otsuka Pharmaceutical Co. Ltd., Yoshitomiyakuhin Corporation, Daiichi Sankyo Company, Limited, Sumitomo Pharma Co. Ltd., Eisai Co. Ltd., Kyowa Pharmaceutical Industry Co. Ltd., Janssen Pharmaceutical K.K., Takeda Pharmaceutical Company Limited, Viatris Inc., Towa Pharmaceutical Co. Ltd., Mitsubishi Tanabe Pharma Corporation, Lundbeck Japan K.K., TSUMURA & Co. and Shionogi & Co. Ltd.

## ETHICS APPROVAL STATEMENT

This study was approved by the Institutional Review Board of Tohoku University (approval number: 2024‐1‐517) and the Medical Ethics Committee of Shinshu University School of Medicine (approval number: 2025‐296).

## PATIENT CONSENT STATEMENT

All participants agreed to provide online informed consent and confirmed their intention to participate in the study.

## CLINICAL TRIAL REGISTRATION

NA.

## Data Availability

The data that support the findings of this study are available on request from the corresponding author. The data are not publicly available due to privacy or ethical restrictions. The JASTIS data are available upon reasonable request via Takahiro Tabuchi.

## References

[1] Bellis MA , Hughes K , Ford K , Ramos Rodriguez G , Sethi D , Passmore J . Life course health consequences and associated annual costs of adverse childhood experiences across Europe and North America: a systematic review and meta‐analysis. Lancet Public Health. 2019;4(10):e517–e528. 10.1016/S2468-2667(19)30145-8 31492648 PMC7098477

[2] Madigan S , Deneault AA , Racine N , Park J , Thiemann R , Zhu J , et al. Adverse childhood experiences: a meta‐analysis of prevalence and moderators among half a million adults in 206 studies. World Psychiatry. 2023;22(3):463–471. 10.1002/wps.21122 37713544 PMC10503911

[3] Madigan S , Thiemann R , Deneault AA , Fearon RMP , Racine N , Park J , et al. Prevalence of adverse childhood experiences in child population samples: a systematic review and meta‐analysis. JAMA Pediatrics. 2025;179(1):19–33. 10.1001/jamapediatrics.2024.4385 39527072 PMC11555579

[4] Merrick MT , Ford DC , Ports KA , Guinn AS , Chen J , Klevens J , et al. Vital signs: estimated proportion of adult health problems attributable to adverse childhood experiences and implications for prevention—25 states, 2015–2017. MMWR Morb Mortal Wkly Rep. 2019;68(44):999–1005. 10.15585/mmwr.mm6844e1 31697656 PMC6837472

[5] Felitti VJ , Anda RF , Nordenberg D , Williamson DF , Spitz AM , Edwards V , et al. Relationship of childhood abuse and household dysfunction to many of the leading causes of death in adults. Am J Prev Med. 1998;14(4):245–258. 10.1016/s0749-3797(98)00017-8 9635069

[6] Felitti V . The relation between adverse childhood experiences and adult health: turning gold into lead. TJP. 2002;6(1):44–47. 10.7812/TPP/02.994 PMC6220625 30313011

[7] Meeker EC , O'Connor BC , Kelly LM , Hodgeman DD , Scheel‐Jones AH , Berbary C . The impact of adverse childhood experiences on adolescent health risk indicators in a community sample. Psychol Trauma Theory Res Pract Policy. 2021;13(3):302–312. 10.1037/tra0001004 PMC8281335 33539157

[8] Osofsky JD , Osofsky HJ , Frazer AL , Fields‐Olivieri MA , Many M , Selby M , et al. The importance of adverse childhood experiences during the perinatal period. Am Psychol. 2021;76(2):350–363. 10.1037/amp0000770 33734800

[9] Senaratne DNS , Thakkar B , Smith BH , Hales TG , Marryat L , Colvin LA . The impact of adverse childhood experiences on multimorbidity: a systematic review and meta‐analysis. BMC Med. 2024;22(1):315. 10.1186/s12916-024-03505-w 39143489 PMC11325707

[10] Gu W , Zhao Q , Yuan C , Yi Z , Zhao M , Wang Z . Impact of adverse childhood experiences on the symptom severity of different mental disorders: a cross‐diagnostic study. Gen Psychiatry. 2022;35(2):e100741. 10.1136/gpsych-2021-100741 PMC9036421 35572774

[11] Lin L , Wang HH , Lu C , Chen W , Guo VY . Adverse childhood experiences and subsequent chronic diseases among middle‐aged or older adults in China and associations with demographic and socioeconomic characteristics. JAMA Netw Open. 2021;4(10):e2130143. 10.1001/jamanetworkopen.2021.30143 34694390 PMC8546496

[12] Myers M , Gumusoglu S , Brandt D , Stroud A , Hunter SK , Vignato J , et al. A role for adverse childhood experiences and depression in preeclampsia. J Clin Trans Sci. 2024;8(1):e25. 10.1017/cts.2023.704 PMC10880014 38384900

[13] Lord C , Elsabbagh M , Baird G , Veenstra‐Vanderweele J . Autism spectrum disorder. Lancet. 2018;392(10146):508–520. 10.1016/S0140-6736(18)31129-2 30078460 PMC7398158

[14] Sasayama D , Kuge R , Toibana Y , Honda H . Trends in autism spectrum disorder diagnoses in Japan, 2009 to 2019. JAMA Netw Open. 2021;4(5):e219234. 10.1001/jamanetworkopen.2021.9234 33944926 PMC8097493

[15] Thomas R , Sanders S , Doust J , Beller E , Glasziou P . Prevalence of attention‐deficit/hyperactivity disorder: a systematic review and meta‐analysis. Pediatrics. 2015;135(4):e994–e1001. 10.1542/peds.2014-3482 25733754

[16] Andrzejewski T , Gomez Batista S , Abu‐Ramadan T , Breitenfeldt KE , Tassone AU , et al. Examining rates of traumatic events and posttraumatic stress disorder symptoms among autistic adults. Autism Adulthood. 2024;6(3):374–387. 10.1089/aut.2023.0022 39371352 PMC11447415

[17] Hartley G , Sirois F , Purrington J , Rabey Y . Adverse childhood experiences and autism: a meta‐analysis. Trauma Violence Abuse. 2024;25(3):2297–2315. 10.1177/15248380231213314 38041427

[18] Kerns CM , Robins DL , Shattuck PT , Newschaffer CJ , Berkowitz SJ . Expert consensus regarding indicators of a traumatic reaction in autistic youth: a Delphi survey. J Child Psychol Psychiatry. 2023;64(1):50–58. 10.1111/jcpp.13666 35817758 PMC10368297

[19] Li ST , Chien WC , Chung CH , Tzeng NS . Increased risk of acute stress disorder and post‐traumatic stress disorder in children and adolescents with autism spectrum disorder: a nation‐wide cohort study in Taiwan. Front Psychiatry. 2024;15:1329836. 10.3389/fpsyt.2024.1329836 38356908 PMC10864464

[20] Okumura K , Takeda T , Komori T , Toritsuka M , Yamamuro K , et al. Adverse childhood experiences exacerbate peripheral symptoms of autism spectrum disorder in adults. Psychiatry Clin Neurosci. 2024;78:580–587. 10.1111/pcn.13712 39037014 PMC11804926

[21] Peterson JL , Earl RK , Fox EA , Ma R , Haidar G , et al. Trauma and autism spectrum disorder: review, proposed treatment adaptations and future directions. J Child Adolesc Trauma. 2019;12(4):529–547. 10.1007/s40653-019-00253-5 31819782 PMC6901292

[22] Rumball F , Happé F , Grey N . Experience of trauma and PTSD symptoms in autistic adults: Risk of PTSD development following DSM‐5 and non‐DSM‐5 traumatic life events. Autism Res. 2020;13(12):2122–2132. 10.1002/aur.2306 32319731

[23] Craig SG , Bondi BC , O'Donnell KA , Pepler DJ , Weiss MD . ADHD and exposure to maltreatment in children and youth: a systematic review of the past 10 years. Curr Psychiatry Rep. 2020;22(12):79. 10.1007/s11920-020-01193-w 33161561

[24] Hadianfard H . Child abuse in group of children with attention deficit‐hyperactivity disorder in comparison with normal children. Int J Community Based Nurs Midwifery. 2014;2:77–84. 25349848 PMC4201192

[25] Stern A , Agnew‐Blais J , Danese A , Fisher HL , Jaffee SR , et al. Associations between abuse/neglect and ADHD from childhood to young adulthood: a prospective nationally‐representative twin study. Child Abuse Negl. 2018;81:274–285. 10.1016/j.chiabu.2018.04.025 29775871 PMC6013278

[26] Ide‐Okochi A , He M , Tokieda T , Nakamura S , Matsunaga N . Assessment of sensory processing issues in children with neurodevelopmental disorders and experiences of maltreatment. Children. 2024;11(2):216. 10.3390/children11020216 38397328 PMC10887822

[27] Sarr R , Spain D , Quinton AMG , Happé F , Brewin CR , Radcliffe J , et al. Differential diagnosis of autism, attachment disorders, complex post‐traumatic stress disorder and emotionally unstable personality disorder: a Delphi study. Br J Psychol. 2025;116(1):1–33. 10.1111/bjop.12731 39300915 PMC11724683

[28] Zhang N , Gao M , Yu J , Zhang Q , Wang W , Zhou C , et al. Understanding the association between adverse childhood experiences and subsequent attention deficit hyperactivity disorder: a systematic review and meta‐analysis of observational studies. Brain Behav. 2022;12(10):e32748. 10.1002/brb3.2748 36068993 PMC9575611

[29] Tokumitsu K , Sugawara N , Tabuchi T , Yasui‐Furukori N . Adverse childhood experiences mediate the association between ADHD symptoms and severe psychological distress in the Japanese general population: a causal mediation analysis of 29,268 participants. BMC Psychiatry. 2025;25(1):1194. 10.1186/s12888-025-07656-9 41310555 PMC12752057

[30] Tabuchi T , Shinozaki T , Kunugita N , Nakamura M , Tsuji I . Study profile: the Japan “Society and New Tobacco” Internet Survey (JASTIS): a longitudinal internet cohort study of heat‐not‐burn tobacco products, electronic cigarettes, and conventional tobacco products in Japan. J Epidemiol. 2019;29(11):444–450. 10.2188/jea.JE20180116 30318495 PMC6776477

[31] Maeda Y , Tabuchi T , Fujiwara T . Association between adverse childhood experiences and pregnancy morbidities: a nationwide online‐based cross‐sectional study. J Obstet Gynaecol Res. 2024;50(12):2231–2238. 10.1111/jog.16124 39414244

[32] Fujiwara T . Impact of adverse childhood experience on physical and mental health: a life‐course epidemiology perspective. Psychiatry Clin Neurosci. 2022;76(11):544–551. 10.1111/pcn.13464 36002401

[33] Kurita H , Koyama T , Osada H . Autism‐Spectrum Quotient‐Japanese version and its short forms for screening normally intelligent persons with pervasive developmental disorders. Psychiatry Clin Neurosci. 2005;59(4):490–496. 10.1111/j.1440-1819.2005.01403.x 16048456

[34] Kessler RC , Adler L , Ames M , Demler O , Faraone S , Hiripi E , et al. The World Health Organization Adult ADHD Self‐Report Scale (ASRS): a short screening scale for use in the general population. Psychol Med. 2005;35(2):245–256. 10.1017/s0033291704002892 15841682

[35] Takeda T , Tsuji Y , Kurita H . Psychometric properties of the Japanese version of the Adult attention‐deficit hyperactivity disorder (ADHD) Self‐Report Scale (ASRS‐J) and its short scale in accordance with DSM‐5 diagnostic criteria. Res Dev Disabil. 2017;63:59–66. 10.1016/j.ridd.2017.02.011 28260624

[36] Prochaska JJ , Sung HY , Max W , Shi Y , Ong M . Validity study of the K6 scale as a measure of moderate mental distress based on mental health treatment need and utilization. Int J Methods Psychiatr Res. 2012;21(2):88–97. 10.1002/mpr.1349 22351472 PMC3370145

[37] Furukawa TA , Kawakami N , Saitoh M , Ono Y , Nakane Y , Nakamura Y , et al. The performance of the Japanese version of the K6 and K10 in the World Mental Health Survey Japan. Int J Methods Psychiatr Res. 2008;17(3):152–158. 10.1002/mpr.257 18763695 PMC6878390

[38] Fujii H , Nishimoto N , Yamaguchi S , Kurai O , Miyano M , Ueda W , et al. The Alcohol Use Disorders Identification Test for Consumption (AUDIT‐C) is more useful than pre‐existing laboratory tests for predicting hazardous drinking: a cross‐sectional study. BMC Public Health. 2016;16(1):379. 10.1186/s12889-016-3053-6 27165437 PMC4862044

[39] Saunders JB , Aasland OG , Babor TF , de la Fuente JR , Grant M . Development of the Alcohol Use Disorders Identification Test (AUDIT): WHO collaborative project on early detection of persons with harmful alcohol consumption‐II. Addiction. 1993;88(6):791–804. 10.1111/j.1360-0443.1993.tb02093.x 8329970

[40] Rigles B . The relationship between adverse childhood events, resiliency and health among children with autism. J Autism Dev Disord. 2017;47(1):187–202. 10.1007/s10803-016-2905-3 27807754

[41] Berg KL , Shiu CS , Acharya K , Stolbach BC , Msall ME . Disparities in adversity among children with autism spectrum disorder: a population‐based study. Dev Med Child Neurol. 2016;58(11):1124–1131. 10.1111/dmcn.13161 27251442

[42] McLaren V , Gallagher M , Hopwood CJ , Sharp C . Hypermentalizing and borderline personality disorder: a meta‐analytic review. Am J Psychother. 2022;75(1):21–31. 10.1176/appi.psychotherapy.20210018 35099264

[43] McCrory EJ , Viding E . The theory of latent vulnerability: reconceptualizing the link between childhood maltreatment and psychiatric disorder. Dev Psychopathol. 2015;27(2):493–505. 10.1017/S0954579415000115 25997767

[44] Infurna FJ , Rivers CT , Reich J , Zautra AJ . Childhood trauma and personal mastery: their influence on emotional reactivity to everyday events in a community sample of middle‐aged adults. PLoS One. 2015;10(4):e0121840. 10.1371/journal.pone.0121840 25849572 PMC4388499

